# Superelastic Behavior of Ti-Nb Alloys Obtained by the Laser Engineered Net Shaping (LENS) Technique

**DOI:** 10.3390/ma13122827

**Published:** 2020-06-23

**Authors:** Damian Kalita, Łukasz Rogal, Piotr Bobrowski, Tomasz Durejko, Tomasz Czujko, Anna Antolak-Dudka, Eduard Cesari, Jan Dutkiewicz

**Affiliations:** 1 Institute of Metallurgy and Materials Science, Polish Academy of Sciences, 25, Reymonta St., 30-059 Krakow, Poland; l.rogal@imim.pl (Ł.R.); p.bobrowski@imim.pl (P.B.); j.dutkiewicz@imim.pl (J.D.); 2 Institute of Materials Science and Engineering, Faculty of Advanced Technology and Chemistry, Military University of Technology, 2, Gen. Kaliskiego Str., 00-908 Warsaw, Poland; tomasz.durejko@wat.edu.pl (T.D.); tomasz.czujko@wat.edu.pl (T.C.); anna.dudka@wat.edu.pl (A.A.-D.); 3 Department of Physics, University of Balearic Islands, E07122 Palma de Mallorca, Spain; eduard.cesari@uib.cat

**Keywords:** additive manufacturing, laser engineered net shaping (LENS), metastable β titanium alloys, Ti–Nb alloys, superelasticity, deformation mechanisms, oxygen effect, mechanical properties

## Abstract

The effect of Nb content on microstructure, mechanical properties and superelasticity was investigated for a series of Ti-xNb alloys, fabricated by the laser engineered net shaping method, using elemental Ti and Nb powders. The microstructure of as-deposited materials consisted of columnar β-phase grains, elongated in the built direction. However, due to the presence of undissolved Nb particles during the deposition process, an additional heat treatment was necessary. The observed changes in mechanical properties were explained in relation to the phase constituents and deformation mechanisms. Due to the elevated oxygen content in the investigated materials (2 at.%), the specific deformation mechanisms were observed at lower Nb content in comparison to the conventionally fabricated materials. This made it possible to conclude that oxygen increases the stability of the β phase in β–Ti alloys. For the first time, superelasticity was observed in Ti–Nb-based alloys fabricated by the additive manufacturing method. The highest recoverable strain of 3% was observed in Ti–19Nb alloy as a result of high elasticity and reverse martensitic transformation stress-induced during the loading.

## 1. Introduction

Ti–Nb-based metastable β titanium alloys are among the most promising candidates for long-term implantation due to their excellent corrosion resistance, biocompatibility and superior mechanical properties [1,2]. Materials for bone implants should fulfill several requirements, among which the most important are appropriate composition, without any toxic elements, and mechanical behavior similar to the bone [3]. These requirements are only partially satisfied with Ti–6Al–4V alloy, commercially used in implantology, in which the alloying elements Al and V can cause allergic reactions and Alzheimer’s disease [3,4]. In addition, an elastic modulus mismatch between cortical bone (10–30 GPa) and the Ti–6Al–4V alloy (100–125 GPa) may result in a stress shielding effect [3,5]. In turn, a low elastic modulus, similar to that of human bone, is observed in Ni–Ti alloys, which additionally exhibit superelastic properties [6]. However, Ni is well known as an allergic and toxic element [3]. The advantages of these two materials (the superior mechanical properties of the Ti–6Al–4V alloy and low elastic modulus and superelastic behavior of Ni–Ti alloys) could be combined in metastable β-titanium alloys while eliminating toxic elements [7,8].

The superelastic behavior observed in β-Ti alloys is associated with a thermo-elastic martensitic transformation between the body centered cubic (BCC) β parent phase and orthorhombic α″ martensite. In a binary Ti–Nb system, alloys containing 26 at.% Nb possess optimal room temperature (RT) superelastic properties, as a result of a martensite start temperature (*M_s_*) slightly below RT [2,9]. Theoretical calculations showed that the maximum transformation strain for the Ti-26Nb alloy is observed along the [011] direction and can reach about 3%. Moreover, this value can be increased even above 4% by a combination of cold-working and aging processes [2]. The superelastic properties of Ti-Nb alloys may also be improved by replacing Nb with other β-stabilizing elements, like Zr, Ta and Mo [9].

The majority of investigations regarding superelastic Ti-Nb alloys were focused on alloys obtained by casting methods [2,7,8,9]. However, nowadays, powder metallurgy (PM) as well as additive manufacturing (AM) are gaining more and more attention due to the possibility of fabricating more complex structural shapes. In addition, porous or cellular structures can be manufactured, which is difficult or even impossible using casting techniques [10,11]. In our previous work [12], we showed that Ti-Nb alloys obtained using mechanical alloying (MA) and spark plasma sintering (SPS) possess a much higher strength in comparison to cast alloys; e.g., the yield strength (YS) of the cast and solution-treated Ti-26Nb alloy reaches about 300 MPa, while, typically, for alloys obtained using the PM route, it exceeds 700 MPa [12,13]. The hardening effect in PM Ti-Nb alloys may be attributed mainly to the effect of interstitial contamination, e.g., oxygen. Kim et al. [14] showed that the ultimate tensile strength (UTS) of the Ti-22Nb alloy increases from about 500 MPa to above 1000 MPa when 2 at.% of oxygen is added. On the other hand, contamination has a significant influence on the superelastic behavior of these alloys. It was confirmed that the addition of 1 at.% of oxygen in binary Ti–Nb alloys leads to a decrease in the *M_s_* temperature by about 160 °C [7]. Lai et al. [15] showed that, in order to obtain RT superelasticity in Ti-Nb alloys, the content of Nb has to be reduced to about 13 at.% to compensate for the influence of oxygen on the martensitic transformation temperatures.

The β-phase Ti-Nb-based alloys have already been fabricated using AM methods. However, powder-bed methods like selective laser melting (SLM) or electron beam melting (EBM) were mainly studied [11,16,17]. In addition, the majority of the works refer to the influence of processing parameters on the microstructure and mechanical properties of materials; e.g., Schwab et al. [18] revealed that the YS and hardness of the SLM-deposited materials increase with energy input as a result of increasing density. Schulze et al. [19] proved that, with the use of SLM, it is possible to obtain a dense material with relatively high density (above 99.5%), and mechanical properties slightly higher in comparison with cast materials but similar to the PM alloys. Instead of using pre-alloyed powders, like in previous works, Wang et al. [20] used a mixture of elemental Ti and Nb powders in order to fabricate Ti-Nb elements. Due to a large difference in the melting points of the metals used, undissolved Nb particles were observed in the microstructure of the as-fabricated elements, which in addition decreased the plasticity of the materials. However, Fischer et al. [21] showed that it is possible to obtain a homogeneous composition by increasing the energy density, even when a mixture of elemental powder is used. Although powder-feed techniques, like laser engineered net shaping (LENS) or direct laser deposition (DLD), are well established in fabricating elements from α or α+β alloys [22,23], there is still limited information concerning β-Ti alloys. It was shown that the microstructure of a Ti-26Nb alloy deposited using laser additive manufacturing (LAM) consists of columnar β grains with strong fiber texture as a result of a large temperature gradient during deposition [24]. Banerjee et al. [25] revealed that the Ti-Nb-Zr-Ta alloy obtained using the LENS technique from elemental powders possesses a low elastic modulus, about 55 GPa, in combination with higher strength in comparison to conventionally fabricated alloys. However, using elemental powders typically resulted in the occurrence of undissolved Nb particles in the microstructure of the fabricated materials [24,25,26].

Despite extensive research regarding the ability to fabricate β titanium alloys using additive manufacturing techniques, there is still no available information in the literature regarding the superelastic effect in those alloys. Taking this into consideration, the aim of this work was to analyze the influence of Nb content on the microstructure, mechanical properties and superelastic behavior of Ti-Nb alloys fabricated using the LENS additive manufacturing method.

## 2. Materials and Methods

### 2.1. Material Fabrication

**Figure 1:**
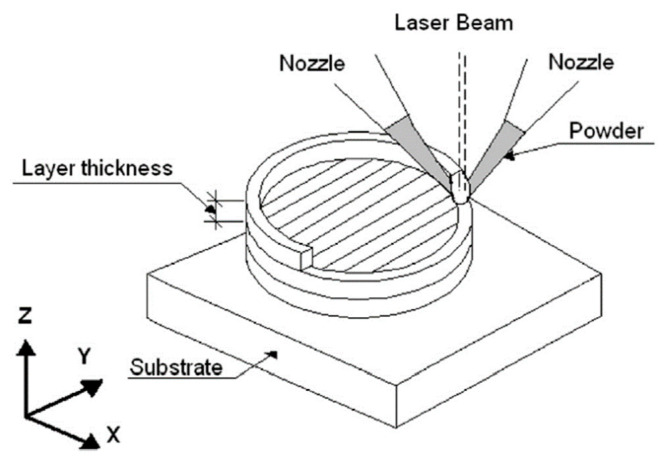
Scheme of the LENS process showing the scanning strategy used. The Z axis indicates the built direction [27].

Elemental Ti (~105 µm, 99.9%) and Nb (~100 µm, 99.9%) spherical powders were used in order to fabricate samples in the form of cylinders 10 mm in diameter and 10 mm in height. The deposition of the Ti–Nb alloys was performed using a LENS MR-7 system (Optomec, Albuquerque, NM, USA), equipped with a 500 W fiber laser and four powder feeders. During deposition, the powders were injected through the nozzles into the melt pool formed on the deposit surface by a highly focused laser beam, as shown in Figure 1 [27]. The LENS process was described in more detail in [28]. The process parameters were as follows: laser power 350 W, laser spot diameter 1.2 mm, overlap 50%, working table feed rate 10 mm/s and single layer thickness 0.3 mm. Five samples, Ti-14Nb, Ti-17Nb, Ti-19Nb, Ti-23Nb and Ti-31Nb (at.%), with different contents of Nb were selected. The flow rates for each elemental powder were estimated based on a calibration that determines the relationship between powder flow rate expressed in [g/min] and feeding rate in RPM (rounds per minute). The chemical composition of the deposited samples was precisely controlled by changing the relative Ti and Nb powder flow rates. The values of the powder flow rate of Ti and Nb for Ti-14Nb, Ti-17Nb, Ti-19Nb, Ti-23Nb and Ti-31Nb samples were: 3.9 and 2.4 g/min, 3.7 and 2.9 g/min, 3.4 and 3.0 g/min, 3.2 and 3.7 g/min, 2.6 and 4.7 g/min, respectively. Due to the high reactivity of the powders with oxygen, the process was conducted in a glovebox under a protective Ar atmosphere (O_2_ ~ 2 ppm). A Ti–6Al–4V plate with a 3 mm thickness was used as the substrate. Solution treatment (ST) was conducted at a temperature of 1250 °C for 24 h, followed by water cooling. To prevent oxidation during annealing, the samples were encapsulated in quartz tubes under vacuum.

### 2.2. Material Characterization

The microstructure of the obtained materials was studied using a Leica DMIRM (Wetzlar, Germany) optical microscope (OM) and an FEI Quanta 3D FEG (Eindhoven, The Netherlands) scanning electron microscope (SEM) equipped with an EDAX Genesis energy-dispersive X-ray spectrometer (EDS). The OM observations were carried out on previously etched samples using Kroll’s reagent. More detailed microstructural observations as well as phase analysis were conducted using a FEI Tecnai G2 F20 (Eindhoven, The Netherlands) transmission electron microscope (TEM). Thin foils for TEM observations were prepared using a Struers Tenupol-5 jet-polisher in an electrolyte consisting of a 10 vol.% of H_2_SO_4_ in methanol at 10 °C. Samples for the compression test were prepared in the form of cylinders 4 mm in diameter and 6 mm in height. Tests were performed using a Shimadzu Autograph AG–X plus (Kyoto, Japan) a testing machine at the strain rate of 10^−3^ s^−1^. In order to determine the superelastic properties, cyclic compressive tests were conducted. In the first cycle, compressive strain reached 1% and then the stress was removed. The tests were repeated for the same sample by increasing the strain by 0.5% for each cycle up to 5%. Tests were carried out at room temperature and three samples for each composition were analyzed. The oxygen content in the analyzed materials was determined by an inert gas fusion technique using a Leco ON836 analyzer (St. Joseph, MI, USA).

## 3. Results and Discussion

### 3.1. Microstructure of the As-Deposited Alloys

**Figure 2:**
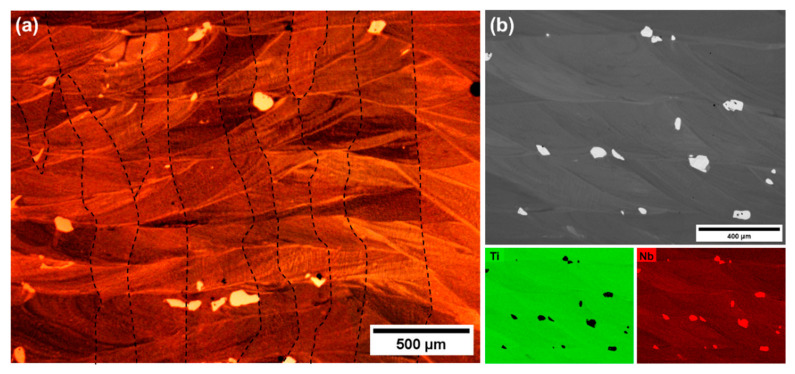
Typical OM microstructure (**a**) and SEM/BSE image, with corresponding EDS chemical composition maps (**b**) of the alloy in the as-deposited state.

Figure 2 shows a typical microstructure of the alloys in the as-deposited state. A layered structure, characteristic for AM processes, formed during the following deposition cycles was observed. The calculated average layer thickness was about 300 µm. Perpendicular to those layers, columnar β grains may be noticed (marked with a dashed line). Their width was in the range between 100 and 500 µm, while their height could reach even a few mm and exceed many times the thickness of a single layer. The grains elongated in the built direction are typically observed in materials fabricated using AM techniques. Their morphology indicates that during the process an epitaxial grain growth in the building direction takes place. This observation has also been confirmed by other authors who analyzed the microstructure of titanium-based alloys obtained using AM techniques [24,25,26]. It is worth mentioning that the applied deposition conditions resulted in the fabrication of almost fully dense materials. The porosity calculated based on micrographs did not exceed 0.15 vol.%. Moreover, microstructural analysis revealed Nb particles undissolved during the process, located mainly at the layer interfaces (Figure 2b). Their presence is related to the relatively high melting point of Nb (2477 °C). This means that the applied energy density of the LENS process was sufficient to melt Ti powder and only partially melt Nb particles, which resulted in a reduction of Nb content in the matrix. The average chemical composition of the matrix at this stage is summarized in Table 1. Nevertheless, the presence of Nb particles in the microstructure of the as-deposited materials was also observed in other works in which elemental Ti and Nb powders were used during a similar process [24,25,26].

It is well known that the chemical composition is one of the most crucial aspects influencing the martensitic transformation temperatures and superelastic behavior. In the case of Nb, the *M_s_* temperature decreases by about 40 °C with 1 at.% on Nb [13]. Therefore, in order to obtain good superelastic properties, the chemical homogeneity has to be high. In the present work, annealing at 1250 °C for 24 h was proposed in order to overcome the inhomogeneity that appeared during the deposition.

### 3.2. Microstructure of Solution-Treated Alloys

**Figure 3:**
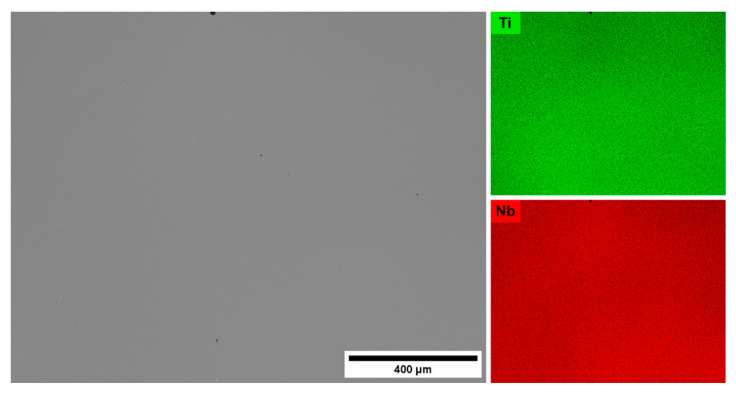
SEM/BSE image and corresponding EDS chemical composition maps of the ST Ti-19Nb alloy.

Figure 3 shows a typical SEM/BSE microstructure and corresponding EDS chemical composition maps of the alloys after solution treatment (ST). It can be noticed that the applied annealing conditions led to the dissolution of Nb particles, giving a homogeneous distribution of the element in the matrix. In addition, no precipitations were observed at this scale. The average chemical compositions of the materials measured using the EDS technique are summarized in Table 2. In order to improve readability in the following sections, the nomenclature based on the Nb content in at.% will be used.

**Figure 4:**
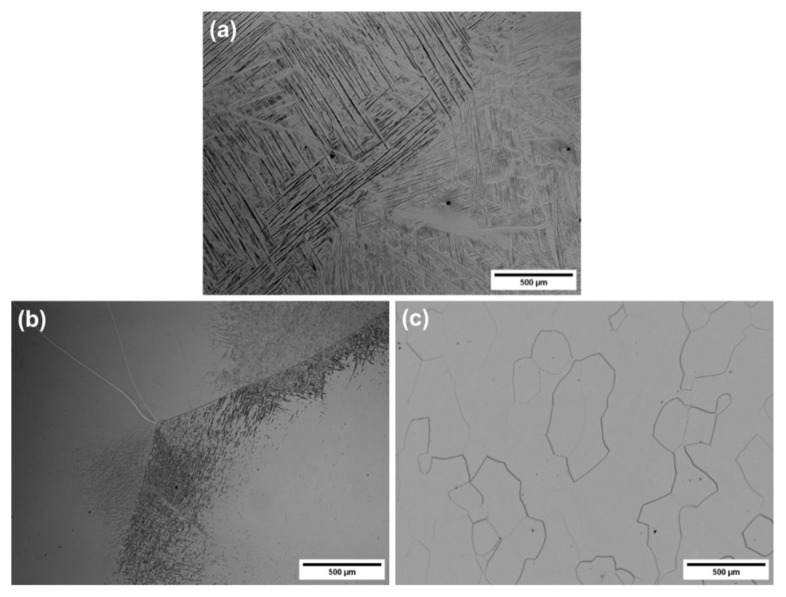
OM microstructures of the ST Ti-14Nb (**a**), Ti-19Nb (**b**) and Ti-23Nb (**c**) alloys.

Figure 4 shows the effect of increasing Nb content on the microstructure of the investigated materials. In the case of the Ti-14Nb alloy, a martensitic microstructure was observed. With further increase in the Nb content, the amount of the martensite decreased, giving a dual-phase β+α″ microstructure for Ti-17Nb and Ti-19Nb and finally a single β phase microstructure for Ti-23Nb and Ti-31Nb. As mentioned above, Nb decreases the *M_s_* temperature (40 °C/at.%) and leads to changes in the α″/β stability [13]. However, in alloys obtained using conventional techniques, like casting, the β phase was observed only above 26 at.% of Nb [2]. The presence of β phase below this value is associated with the effect of interstitial contamination, mainly oxygen. Typical oxygen content in cast Ti-Nb-based alloys is below 0.5 at.% [29,30]. Meanwhile, a high oxygen level, in the range of 1.5–4.0 at.%, was observed in titanium alloys obtained using powder metallurgy techniques, which is associated with a high tendency of titanium powders to oxidize [15,31]. In this case, the measured oxygen content was 2 at.% for all of the investigated materials. Oxygen significantly suppresses the martensitic transformation in the Ti–Nb alloys and decreases the transformation temperature by about 160 °C/at.% [14]. Taking this into consideration, to compensate for the effect of oxygen and to maintain the transformation temperature at a constant level, Nb content should be reduced by about 8 at.%. This is in agreement with the obtained result—the Ti-19Nb alloy shows only traces of martensite at RT (Figure 4b). Similar behavior was observed in the Ti-Nb alloys prepared by sintering, e.g., Lai et al. [15] showed that Nb content has to be reduced to 14 at.% in order to decrease the *M_s_* temperature below RT in the alloy containing about 3 at.% of oxygen.

**Figure 5:**
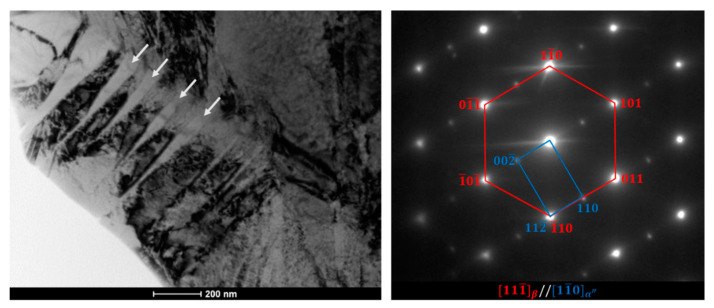
BF-TEM image and corresponding SADP of ST Ti-19Nb.

Figure 5 shows a bright-field (BF) TEM image of the ST Ti-19Nb alloy. In the microstructure of the alloy, parallel α″ needles, marked with arrows, with a width of tens of nanometers can be distinguished in the β phase matrix. This is in agreement with the OM microstructure (Figure 4b), which indicates that, during the quenching, only partial transformation took place. The corresponding selected-area diffraction pattern (SADP) registered along the ${\left[11\bar{1}\right]}_{\beta }$ zone axis revealed weak reflections corresponding to the orthorhombic structure of α″ martensite. The following orientation relationship was observed between the martensite and parent phase: $[1\bar{1}0{]}_{\alpha^{\prime \prime }}//[11\bar{1}{]}_{\beta }$. This has also been reported by other authors [32].

**Figure 6:**
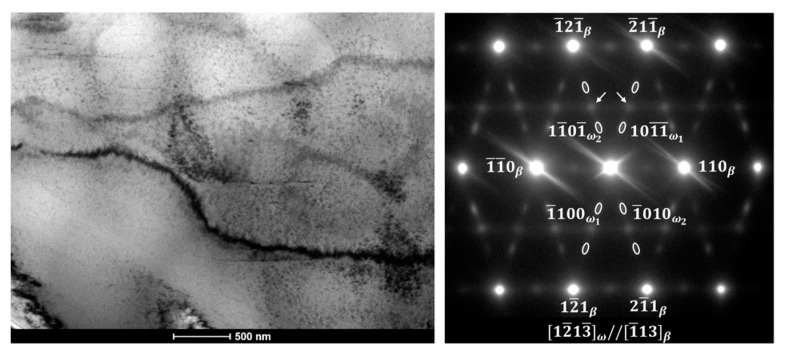
BF-TEM image and corresponding SADP of ST Ti-23Nb.

Figure 6 shows the BF-TEM image and corresponding SADP of the ST Ti-23Nb alloy. In addition to primary reflections from the β matrix with the ${\left[\bar{1}13\right]}_{\beta }$ zone axis, diffused scattering corresponding to a hexagonal ω phase at 1/3 and 2/3 ${\left\{1\bar{2}1\right\}}_{\beta }$ was observed. Two types of ω phase may be distinguished according to the formation mechanism—an athermal ω phase (ω_ath_), which forms during quenching by the displacive mechanism, and an isothermal ω phase (ω_iso_), which appears at intermediate annealing via the diffusion mechanism [33,34]. In this case, samples were quenched from the β phase field; thus, reflections are associated with the ω_ath_ phase. Formation of the ω_ath_ involves the collapse of a pair of (222)_β_ planes to an intermediate position, which results in four crystallographic variants. The presented SADP shows only two variants, while two more contribute to the β reflections [35]. The found orientation relationship was $[\bar{1}13{]}_{\beta }//[1\bar{2}1\bar{3}{]}_{\omega }$. Although the ω phase is metastable under ambient conditions, it is typically observed in Ti-Nb alloys in the form of nanometric precipitations uniformly distributed in the matrix [12,36,37]. The presence of these precipitations mechanically suppresses the martensitic transformation, but, on the other hand, it increases the critical stress for slip deformation which is low for single-phase Ti-Nb alloys [2]. More detailed studies revealed that, during the deformation, the ω phase may be transformed to the α″ martensite [38,39]. Additional diffraction spots, marked with arrows, correspond to the nano-domains (O’) which are commonly observed in oxygen-added Ti-Nb-based alloys [29,40].

### 3.3. Mechanical Properties

**Figure 7:**
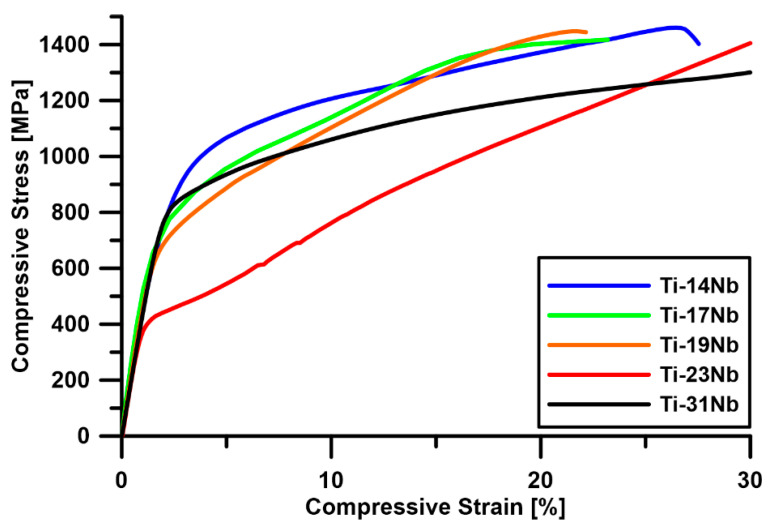
Compressive strain–stress curves for a series of Ti-xNb alloys in the ST state.

**Figure 8:**
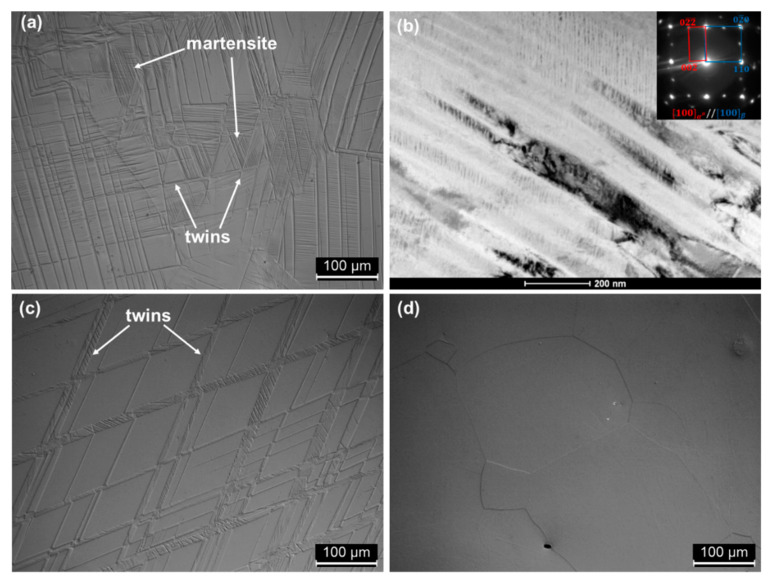
OM microstructures of deformed, up to 5% in compression mode, Ti-19Nb (**a**), Ti-23Nb (**c**) and Ti-31Nb (**d**) alloys. The BF-TEM image and corresponding SADP of deformed Ti-19Nb alloy (**b**).

In order to determine the mechanical properties of the fabricated materials, compression tests were carried out. Figure 7 shows the compression stress–strain curves for a series of Ti-xNb alloys in the ST state. The results are summarized in Table 3. High YS, of about 730 MPa, was observed in the case of the Ti-14Nb alloy. With the addition of Nb, the YS decreases to 420 MPa for the Ti-23Nb alloy and then increases to 760 MPa for Ti-31Nb. The deformation behavior of metastable β phase titanium alloys is complex and highly dependent on the β phase stability, which is a function of the alloy composition. In general, with increasing amounts of β stabilizing elements, the deformation mechanisms follow the sequence: martensite (α″) deformation → stress-induced martensitic transformation (SIMT) (β→α″) → twinning → slip [41,42]. It is worth noting that, typically, these mechanisms overlap, so the occurrence of a single mode is rather rare in metastable alloys. Taking this into account, the observable changes in YS may be attributed to changes in the deformation modes. Ti-14Nb is in a martensitic state at RT; thus, the deformation of this phase must control the mechanical properties of the alloy. However, with increasing Nb content, the stability of the β phase increases and the amount of the orthorhombic α″ decreases. As a result, YS decreases to 700 and 670 MPa in the case of Ti-17Nb and Ti-19Nb, respectively. Figure 8 shows the microstructures of the deformed samples in the compression mode up to 5%. It can be noticed that two deformation modes occurred during the loading of Ti-19Nb: SIMT and twinning. Microstructural observations of the deformed alloy revealed the presence of plate-like twins and needle-like α″ martensite between the shear bands. The occurrence of a high density of twinned martensite needles was also confirmed using TEM—Figure 8b. It is important to mention that some amount of the martensite may, in addition, transform to the parent phase during unloading as a result of a reverse transformation. With further addition of Nb, the *M_s_* temperature decreases, leading to a single β phase microstructure of Ti-23Nb. The deformation of this alloy occurs mainly through the twinning mechanism. In the deformed microstructure (Figure 8c), quite large shear bands can be observed. Visible inside the twin lamellar structure may be both secondary twins or stress-induced α″ [43]. More important evidence of twinning deformation in Ti-17Nb, Ti-19Nb and Ti-23Nb arises from the shape of the stress–strain curves. The twinning mechanism is typically associated with a high work-hardening rate during deformation. In the case of these three alloys, the slope of the curves in the plastic range is much higher in comparison to Ti-14Nb and Ti-31Nb, in which different mechanisms were observed.

The twinning mechanism of deformation has frequently been observed in the metastable β titanium alloys. Typically this is associated with a low YS between 200 and 500 MPa, e.g., in Ti-24Nb-4Zr-8Sn (~200 MPa) [44], Ti-12Mo (~480 MPa) [43], and Ti-9Mo-6W (480 MPa) [45], a high work-hardening rate and large uniform elongation. This is in agreement with the obtained results—the YS of Ti-23Nb was about 420 MPa, and no fractures were observed during the deformation up to 50% in the compression mode. There are two twinning systems occurring in the β titanium alloy depending on the β stabilizing elements: {112} <111> and {332} <113> [46]. In the binary Ti–Nb system, twinning was observed in alloys with Nb content higher than 26 at.% (the required Nb content to obtain β phase at RT) [41]. In our case, twinning was observed when Nb content exceeded 17 at.%. This can be attributed to the high oxygen content, about 2.0 at.%, which arose from the fabrication method. Oxygen is considered an α stabilizer in titanium alloys. However, in the case of β-Ti alloys, oxygen promotes stress-induced mechanisms similarly to the β stabilizers [41,42]. In order to determine the phase stability in β phase titanium alloys, a $\bar{B_{o}}-\bar{M_{d}}$ (bond order—d-orbital energy level) diagram could be used. Abdel-Hady et al. [42] showed that the oxygen addition shifts the single β phase boundary to the region of the lower amount of β stabilizers. Simultaneously, SIMT and twinning deformation mechanisms may be observed in alloys containing lower levels of β-stabilizing elements when they contain oxygen.

Further increasing of β phase stability, as a result of Nb addition, causes an increase in the strength of the Ti-31Nb alloy. Figure 8d, showing the deformed microstructure of this alloy, does not present any evidence of stress-induced transformations. This indicates that the deformation occurs through the slip mechanism. This is in agreement with the literature; e.g., Min et al. [47] showed that the YS of the Ti-15Mo (wt.%) alloy increases from 439 MPa to 837 MPa by adding 1 wt.% of Fe, as a result of the change in the deformation mode from twinning to slip. The slip is typically observed in highly stabilized alloys like Ti-25Ta-20Zr or Ti-30Zr-10Nb-10Ta [42]. These alloys exhibit high YS, as well as a low work-hardening rate. The critical Nb content is about 32–36 at.% for the binary Ti–Nb system [41]. In this case, the required Nb content to activate the slip mechanism was slightly lower as a result of the high oxygen content, as described previously.

Table 3 also lists the values of the elastic moduli (*E*) for the investigated alloys. Typical values of *E* for metastable β titanium alloys are in the range between 100 GPa and 50 GPa [48]; however, even lower values are observed in alloys containing high oxygen levels, e.g., below 30 GPa in sintered Ti-(8-18)Nb alloys [15]. In this case, the lowest modulus, of about 40 GPa, was observed for the Ti-19Nb alloy. Generally, the elastic modulus of the phases observed in titanium alloys increases in the sequence β < α″ < α < ω [49,50]. Taking this into consideration, the elastic modulus first decreases with Nb content as a result of the decreasing amount of α″ martensite. However, the occurrence of ω phase precipitations in the case of alloys containing a higher amount of Nb resulted in an increase in this value.

### 3.4. Superelastic Behavior

Cyclic compressive tests were carried out in order to determine the superelastic properties of the investigated alloys. The total recoverable strain (*R_T_*) is composed of three parts: the recovery of its own elasticity (*R_E_*), the elastic recovery of pore structure (*R_P_*), and recovery associated with reversible martensitic transformation (*R_MT_*)—Equation (1) [15]:

(1) $$R_{T}=R_{E}+R_{P}+R_{MT}$$

**Figure 9:**
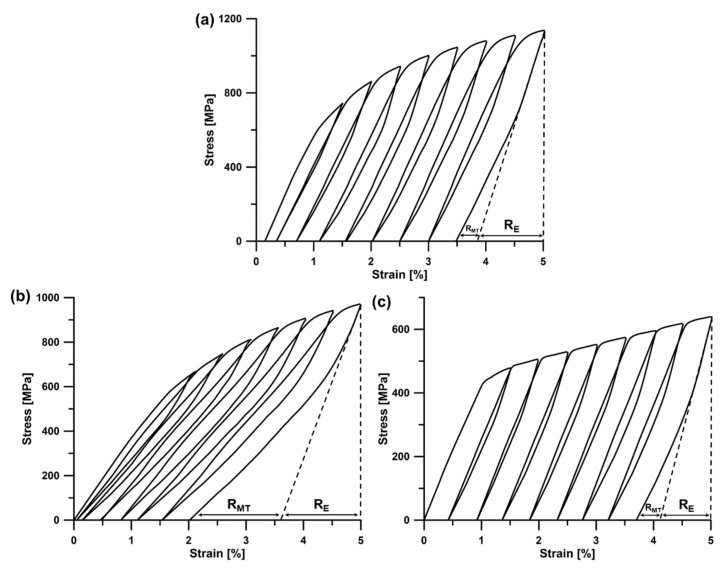
Cyclic compressive stress–strain curves of the ST Ti-14Nb (**a**), Ti-19Nb (**b**) and Ti-23Nb (**c**) alloys.

The porosity of the fabricated materials was low (did not exceed 0.15 vol.%); thus, the *R_P_* part can be neglected. As a result, *R_T_* depends mainly on the *R_E_* and *R_MT_* parts. Figure 9 shows typical examples of the cyclic stress–strain curves, alongside the method used in order to determine the *R_E_* and *R_MT_* values. The obtained results are summarized in Table 4.

The superelastic effect in Ti-Nb-based alloys is associated with the reversible stress-induced martensitic transformation β↔α″. However, SIMT may occur only in a specific temperature range—above *A_f_* (or above *A_s_*, but then only partial recovery is observed), but below *T_s_* (this describes the temperature above which other deformation mechanisms are observed).

The measured recoverable strain reaches about 1.5% for the Ti-14Nb alloy. As follows from Figure 9a, this value mainly comes from the *R_E_* part, which is related to the martensitic microstructure of the alloy (*M_s_* has to be below RT). However, with increasing Nb content, the transformation temperature decreases and the β phase becomes more stable. This causes an increase in the value of the *R_MT_* part in the case of the Ti-17Nb and Ti-19Nb alloys for which SIMT can be observed. The maximum recoverable strain, as high as 3%, was observed for the Ti-19Nb alloy (Figure 9b). Taking into consideration that in the microstructure of the alloy only traces of martensite were observed, the *A_f_* temperature should be slightly above RT. Further addition of Nb caused a decrease in recoverable strain as a result of a change in the deformation mode from SIMT to twinning and slip as described in more detail in Section 3.3. The relatively high values of *R_E_* of the investigated alloys are associated with their low elastic modulus and high YS (Table 3).

It is important to note that the superelastic behavior of the Ti-19Nb alloy (Figure 9b) is different in comparison to the materials obtained using conventional techniques. The differences arise from the elevated oxygen content observed in the investigated materials. During loading of an oxygen-free superelastic alloy typically two-stage yielding is observed—the first yielding point represents the critical stress for SIMT (100–200 MPa for binary Ti–Nb alloys) and the second point is associated with the permanent deformation [2,13]. On the other hand, only one-stage yielding is observed in alloys containing the appropriate amount of interstitial elements, similarly to Ti-19Nb [14,51]. This non-conventional behavior is associated with the suppressing effect of oxygen on the martensitic transformation. The phenomenon of the superelasticity in oxygen-rich alloys has not yet been fully explained. There are a few mechanisms proposed in the literature. Castany et al. [52] showed that the superelasticity of the Ti–24Nb–0.5O alloy involves the combination of a wide elastic deformation of the parent phase (up to about 2% of strain) and reversible SIMT. Moreover, tensile tests under synchrotron X-ray radiation revealed that the critical stress for SIMT is similar to the YS of the alloy. On the other hand, Tahara et al. [29] found that long-range martensitic transformation is suppressed by the formation of nano-sized lattice modulations in the alloys which contain oxygen. In this case, instead of SIMT the superelasticity results from the growth of the favorable domains during the deformation which can return to the initial state during unloading [29].

The obtained recoverable strain for Ti-19Nb is slightly higher in comparison to cast Ti-Nb binary alloys in the solution-treated state. Kim et al. [2] reported 2.5% of recoverable strain in Ti–26Nb, similarly to Tahara et al. [53]. A lower value, of about 2%, for the same alloy was observed by Kim et al. [13]. However, due to a low critical stress for SIMT in those alloys, e.g., about 175 MPa [13] or 100 MPa [2], the *R_MT_* to *R_T_* ratio is higher in comparison to the investigated Ti-19Nb. The higher values of recoverable strain were observed in the cast alloys in the aged state, e.g., 4.2% for Ti-26Nb aged at 400 °C for 1h [2] or 3.5% for the Ti-26Nb alloy cold rolled and aged at 600 °C for 6 min [54]. On the other hand, high recoverable strain, up to 5%, was observed in the sintered Ti-13Nb alloy [15]. This value was associated mainly with the *R_E_* part, as a result of the low elastic modulus (25 GPa) and high YS (above 1000 MPa). In our previous work [12], we also showed 3% of recoverable strain for sintered Ti-14Nb alloy, however, this value was related mainly to the *R_E_* part because of the high YS (920 MPa) of the alloy.

As far as the authors are aware, the superelastic effect has not been observed in β phase titanium alloys obtained using AM techniques until now. The vast majority of the investigation in the field of AM concerns the influence of the processing parameters on the properties and microstructure of the alloys [18,55,56] or the fabrication of porous structures [11,57]. Fischer et al. [58] conducted cyclic tests on Ti-27.5Nb fabricated using the CLAD technique but no evidence of superelasticity was reported. The presented results revealed that it is possible to obtain relatively good superelastic properties, induced at a high stress near 700 MPa, in alloys fabricated using AM techniques; however, it is necessary to include the effect of oxygen contamination during the development of the alloy composition.

## 4. Conclusions

The influence of Nb content on microstructure, mechanical properties and superelastic behavior was studied for a series of Ti-xNb alloys fabricated using the laser engineered net shaping process. Based on the experimental observations, the following conclusions were drawn:

- The applied LENS fabrication parameters allowed the fabrication of almost fully dense materials with porosity of about 0.15 vol.%. However, the energy density was insufficient to melt the Nb particles. Additional annealing at 1250 °C for 24 h was necessary in order to ensure a suitable level of homogeneity.
- The phase composition of the investigated alloys changed from single α″ martensite (Ti-14Nb) to a mixture of α″ + β phases (Ti-17Nb, Ti-19Nb) and finally β + ω_ath_ (Ti-23Nb, Ti-31Nb).
- The observed changes in the mechanical properties of the investigated materials were associated with changes in the deformation modes. The SIMT and twinning mechanisms were observed in the Ti-17Nb and Ti-19Nb alloys, but it should be noted that with increasing Nb content the mechanism evolved to twinning in Ti-23Nb and slip in Ti-31Nb. Similar behavior was observed in cast alloys; however, in our case, the specific mechanism occurred at lower Nb concentration, which was associated with higher oxygen content. This leads to the conclusion that, although oxygen is considered as an α stabilizing element, it increases β phase stability in the β phase alloys.
- For the first time, superelastic behavior was observed in Ti–Nb alloys obtained using additive manufacturing techniques. The maximum recoverable strain, as high as 3%, was registered in the Ti-19Nb alloy. The obtained results allowed the statement to be made that oxygen has a significant influence on the superelasticity of alloys fabricated using AM techniques, which should be considered during the development of new compositions.

## Figures and Tables

**Table 1:** Chemical composition of the matrix in the as-deposited state.

Chemical Composition	Sample				
	1	2	3	4	5
Ti [at.%]	90.2	89.0	87.9	85.7	80.5
Nb [at.%]	9.8	11.0	12.1	14.3	19.5

**Table 2:** Average chemical composition of the ST alloys.

Chemical Composition	Sample				
	1	2	3	4	5
Ti [at.%]	85.8	82.1	80.7	77.2	69.2
Nb [at.%]	14.2	17.2	19.3	22.8	30.8

**Table 3:** Mechanical properties of the ST Ti-Nb alloys.

	Alloy Composition [at.%]				
	Ti–14Nb	Ti–17Nb	Ti–19Nb	Ti–23Nb	Ti–31Nb
YS [MPa]	734 ± 12	695 ± 38	669 ± 36	418 ± 19	762 ± 22
A [%]	21 ± 3	23 ± 10	22 ± 4	> 50	> 50
E [GPa]	54 ± 6	49 ± 5	40 ± 6	49 ± 6	55 ± 2

**Table 4:** Total recoverable strain (*R_T_*) divided into recoverable strain associated with alloy elasticity (*R_E_*) and reversible stress-induced martensitic transformation (*R_MT_*) for the investigated materials.

	Alloy Composition				
	Ti–14Nb	Ti–17Nb	Ti–19Nb	Ti–23Nb	Ti–31Nb
*R_T_* [%]	1.5	2.0	3.0	1.3	1.6
*R_E_* [%]	1.2	1.2	1.4	0.9	1.4
*R_MT_* [%]	0.3	0.8	1.6	0.4	0.2
